# Building the workforce to care for the aged: Can accreditation contribute?

**DOI:** 10.3389/fpubh.2022.1062469

**Published:** 2022-11-10

**Authors:** Connie J. Evashwick

**Affiliations:** Milken Institute School of Public Health, George Washington University, Washington, DC, United States

**Keywords:** workforce to care for the aged, geriatrics workforce, shortage of geriatric specialists, educating the health professions workforce for geriatrics, accreditation of geriatric education programs, health professions education accreditation requirements

## Building the workforce to care for the aged: Can accreditation contribute?

As the world's population ages, the workforce needed to care for older people is challenged in capacity and competence. Simply put, the world does not, at the present time, nor will it in the near future, have enough clinicians, support professionals, or caregivers to meet the complex and multi-faceted needs of the 1.6 billion people who will be aged 65 and above by 2050 (1). This paper explores the role that educational accreditation agencies could play in increasing the training of the workforce to care for the aged.

## Matching need and supply through education

The rapid growth in the number of people aged 65 and older around the globe and in individual countries is well–documented (2). Among the older population, the number of people suffering from Alzheimer's disease and related dementias is also increasing (3). The numbers are relevant as they indicate the magnitude of the challenge to recruit and train the workforce required to care for older people, especially those with complex chronic medical conditions, orthopedic limitations, and mental decline, among other multi-faceted problems. Compared to the need, clinicians trained in geriatrics are in short supply (4).

In an ideal system, demand—represented by the increased number of older people, modified by some index to indicate complex healthcare needs—would be accommodated by supply—represented as jobs available and filled. Higher education would be directly related to employment, providing the training required of students to get the jobs available. Yet, in many countries in the world, the systems of higher education and employment are not directly related, accrediting agencies interject diversions, and demand for health and related services is distorted by payment systems, gatekeepers, and culture, among other issues. The argument proposed in this paper is that accreditation of university health professions educational programs is one intervention, overlooked as of now, that could bridge the disconnect between education and a much-needed workforce.

## Training of clinical geriatricians

Despite the work of early pioneers on the special medical needs of older adults, recognition of geriatric medicine as a clinical specialty began in earnest only in the first half of the 20^th^ century. The first faculty chair in geriatrics was established in 1965 in the United Kingdom (United Kingdom) (5). In the late 1970s, the United States (United States) made a major thrust to incorporate geriatrics into medical education and to establish advanced training programs (i.e., residencies and fellowships) in geriatric medicine (6). Other countries in Europe and throughout the world followed over the ensuing decades. The Malaysia Society for Geriatric Medicine, for example, prepared a comprehensive report in 2019 about the need for expanded efforts to meet the impending increase in the aged in Malaysia (7). Dentistry, too, began in the 1970s to examine the need for educating dental students to care for older adults. Brazil established the first geriatric dentistry specialty in 2010 (8).

Fast forward to the third decade of the 21^st^ century, and the results of the wizened call for education of physicians, dentists, nurse practitioners, social workers, pharmacists, physical therapists, occupational therapists, and other health professionals who specialize in geriatrics are meager. The United Kingdom, the early leader in geriatric medicine education, reports having 1,747 consultant geriatricians, far less than the projected number needed if applying the approximate standard of 1 consultant per 800 older adults (9). The United States has about 7,100 geriatricians as of 2022 (10), but estimates 30,000 will be needed by 2025 (11). Australia, which projects that its population age 65 and older will increase from 16% of the total population in 2020 to 21–23% by 2066 (12), reported having 874 geriatricians in 2019, to serve a total nationwide older population of 4.2 million (13). Malaysia projects that its population age 60 and older will double from 7% of the country's total population in 2019 to 14% by 2040, or about 8.2 million people. Malaysia reports having only 40 geriatricians in 2019 (14). Dentistry has achieved a rate of education of about two-third of schools worldwide offering mandatory geriatric dentistry courses, but nonetheless questions if this is sufficient to meet the needs of the expanding number of older adults (15). These sample numbers and discipline-specific studies make it evident that the world will not produce enough geriatric specialists to meet the complex needs of the expanding aged population.

This shortage of specialists then leads us to assert that all health professionals should have at least a basic understanding of geriatrics. This would improve quality of care, spread the work, and use those who do have advanced training to care for older adults with the most complex conditions, as well as to lead the systemic changes that must be taken over the longer term to bring capacity closer to the need and demand for services.

## Geriatrics education and accreditation

We would propose to incorporate geriatrics into the education of all health professions students, in all disciplines, at all stages of training. Except for countries where the governmental controls the content of education, the means to do so would be through requirements of the agencies that accredit health professions training programs. This does not take government action or new resources; it takes societal recognition of the importance of such training and commitment by the health professions and, in particular, the faculty and mentors who train today's students.

The majority of post-high school educational programs in developed countries, as well as many in developing countries (16), are driven by accreditation by an external body. Evidence suggests that, at the present time, accrediting agencies do not highlight care of the aged as an essential element of the education of a health professional. Although exceptions exist, this lack of attention to care of the aged seems to be pervasive across all disciplines in most countries. Educational accrediting agencies have power over university programs, their numbers are small enough to focus an effort for change, and the content of their work emanates from members of the field. These are conditions amenable for an advocacy campaign to succeed. Once well-known universities begin to change, others follow, whether forced by accreditation or a desire for quality or a business goal of being competitive.

One caveat is that accrediting requirements are often broad, allowing the university and faculty considerable leeway in constructing a curriculum and courses appropriate for their target audience of students and the local employment context. However, we have also seen that specific topics can be incorporated when deemed important by society. Recent examples include expectations for diversity, equity, and inclusion (DEI), social determinants of health (SDOH), and climate change. Why not geriatrics?

## Current accreditation requirement content

If this were a research project, we would start with two questions: (1) Do the agencies that accredit health professions education include geriatrics among the topics required within a curriculum? And (2) If geriatrics is found to be required by accrediting agencies, does it make a difference in the expertise of health professionals in geriatrics and/or in the percentage of clinicians who choose to specialize in geriatrics? In preparation for a more extensive study, we conducted a pilot project to examine the criteria used by a variety of health professions accrediting agencies for different disciplines in various countries. We acknowledge that the examples do not represent any type of statistically valid sample. Nonetheless, the results are revealing. Select examples follow.

In the United States, the Council on Education for Public Health (CEPH) specifies the “foundational knowledge” required of all undergraduates and graduate students enrolled in degree programs granted by schools or programs of public health, as well as competencies required of master's and doctoral level students (17). CEPH specifies some content and competencies in great detail. Master's content areas include biological, genetic, behavioral, psychological, social, political, and economic factors the affect health, and for undergraduate knowledge, “opportunities for promoting and protecting health across the life course.” Neither undergraduate nor graduate public health students are required to know specifically about aging. In contrast, a graduate-level competency specifically focuses on “racism.” With regard to other demographics, and specifically aging, however, CEPH is silent. With about 25,000 new enrollees per year in public health graduate programs alone, many more students could become aware of the conditions faced by the aging population.

Nursing basic education may incorporate training about the care of older adults, but it lacks a clear directive to focus on older adults as a priority. The Accreditation Commission for Education in Nursing, which accredits nursing education programs in the United States, clearly avoids any specification of content (18). Nonetheless, its Standards do include topics deemed important, such as “health literacy” and “use of technology.” The National Council Licensure Examination (NCLEX) is taken by all nursing program graduates in the United States. Accredited schools typically prepare their students to take and pass the exam. The exam has eight content categories organized by clinical topic. Although questions about older adults might be scattered throughout the exam, no specific instructions alert the nursing student or their professors that students should know about geriatrics.

The Japan University Accreditation Association accredits six types of university education programs and publishes standards, including Dental Education Standards (19). Although Japan has one of the highest proportions of aged populations in the world, no mention is made of the need to incorporate knowledge of geriatrics into the dental curriculum.

The Australian Physiotherapy Council accredits Australian higher education entry-level programs in physical therapy (20). Program of Study Requirement 3.3 and Foundational Ability “C” include knowledge of clients “across the life span,” but do not call out geriatrics per se.

In Ghana, the National Accreditation Board accredits university programs in the health professions disciplines of medicine, nursing, pharmacy, dentistry. For nursing, all curricula and exams are regulated by the Nurses and Midwifery Council of Ghana (21). After 3 years of basic education, students are eligible to pursue specialties, which include community nursing, ophthalmologic nursing, pre-operative nursing, ENT nursing, public health nursing, critical care nursing, nurse anesthesiology, and community oral health—but not geriatrics or chronic illness care.

## Accreditation requirement vagueness

When geriatrics is included in curricula, the content and extent of training can be highly varied. Physicians in training in the United States are expected to meet the Minimum Geriatric Competencies for Medical Students (22), but how these are taught is left to each School of Medicine to weave into its curriculum. One well-recognized program requires students to spend 1 month doing an in-person clinical rotation in Pediatrics, a separate in-person rotation on OB/GYN delivery, and 1 week doing a virtual course on Geriatrics. The content is there, assessed by an on-line exam, but the proportion is inconsistent with the demographics of most patient panel populations.

A recent study comparing geriatric dentistry curricula across six continents found that geriatric dentistry was a mandatory course in more than two-thirds of the 83 responding dental schools representing 24 countries (23). Similarly, about two-thirds had mandatory clinical rotations in geriatric dentistry. Other schools had elective courses in geriatric dentistry. Differences were not explained by type of school, location, or method of teaching. That means that one-third of dental schools *do not* have geriatrics in didactic or clinical training and those that do exhibit wide variation.

## Discussion

Research shows that students are more likely to choose a career path if they have had exposure to it through their life or education experiences. Incorporating basic education about geriatrics into the curricula of all health professions disciplines could be one means to increase access by older adults to appropriate health care and related services. Entities that accredit colleges, universities, and other educational programs could drive changes to health professions curricula to incorporate geriatrics. This should not be a token inclusion, but a comprehensive, in-depth, practice as well as theoretical, addition to academic and applied training.

Moreover, agreement among and across accrediting agencies could produce a consistency in baseline education across disciplines. A shared baseline knowledge and a realistic expectation of how other disciplines have been trained would lead to more effective interdisciplinary teams to deal with the multi-faceted clinical needs of older adults.

How does one control accrediting agencies? The majority of accrediting bodies, whether focused on educational curricula or competencies required of an individual for licensing or certification, are comprised of health professionals working in a given field. Expert panels are brought together to delineate subject matter and competencies. Recommendations are vetted among more professionals from the same discipline and at times across disciplines. Content is driven by the latest evidence from the field, not by commercial or personal interests. Conceptually, then, it should be possible to educate those involved in writing the accreditation “standards” of the importance of addressing the aging of the world's population and the rationale for including baseline content. Striving for compatibility or harmony across disciplines would lend even greater perspective and perhaps a wide array of educational self-study tools that span disciplines.

Critics might argue that this approach is naïve and unrealistic. We would counter that when society embraces wide-spread acceptance of a problem, a solution is possible. Despite more than 50 years of trying to build a workforce of expert geriatricians, the world and most individual countries have failed to do so. Those involved with the care of older adults must exert leadership and launch a serious initiative for awareness and change.

## Conclusion and next steps

Myriad factors inhibit providing an adequate supply of well-trained health professionals to meet the growing, multi-faceted demand of an aging population. The above argument is offered as one suggestion to an extremely complex, recalcitrant problem. Next steps from an academic perspective would be a comprehensive geriatric workforce analysis: an enumeration of available specialists by discipline, by country or region; a comparison to the current and future demand of the aging population; a detailed analysis of educational programs, competency models, accrediting entities, and criteria for accreditation of university education programs, as well as certification and licensure for individuals. These tasks alone cannot solve the shortage in the workforce to care for the aged that relate to poor system infrastructure, low salaries, geographic maldistribution, student preferences, or cultural differences. Nonetheless, they can provide some insights into one path that might lead to progress over the long-term. Following a Stages of Change model, making care of the aged a well-known, well-publicized international problem is the first step toward future improvement, and starting with increasing the awareness of health professionals through accreditation requirements may be a good beginning.

## Author contributions

This manuscript was conceived of and written by CE.

## Conflict of interest

The author declares that the research was conducted in the absence of any commercial or financial relationships that could be construed as a potential conflict of interest.

## Publisher's note

All claims expressed in this article are solely those of the authors and do not necessarily represent those of their affiliated organizations, or those of the publisher, the editors and the reviewers. Any product that may be evaluated in this article, or claim that may be made by its manufacturer, is not guaranteed or endorsed by the publisher.
